# Large-vessel vasculitis in graft-versus-host disease: a case report

**DOI:** 10.1186/s13256-021-03067-y

**Published:** 2021-09-28

**Authors:** Anmar Al-Heilfi, Champa Nataraja, Helen Cooley, Tracey Batt

**Affiliations:** Royal Hobart Hospital, Hobart, TAS Australia

**Keywords:** Graft-versus-host disease, Large-vessel vasculitis, Allogenic stem cell transplant

## Abstract

**Background:**

Graft-versus-host disease is a common complication seen with allogenic stem cell transplant, which is used to treat a variety of hematological malignancies. Graft-versus-host disease is an allogenic syndrome and can present in a variety of ways, including symptoms mimicking various autoimmune diseases; however, it is quite rare to see graft-versus-host disease affecting the vascular system and causing vasculitis.

**Case presentation:**

We describe a case of a 59-year-old Caucasian man with follicular lymphoma and diffuse large B-cell transformation who developed graft-versus-host disease post allogenic hematopoietic stem cell transplantation and later progressed to neurological complication foot drop and large-vessel vasculitis.

**Conclusion:**

The life-threatening vascular complications associated with large-vessel vasculitis include arterial aneurysms and dissections, and ischemic or hemorrhagic stroke. Thus, this rare immunological association needs to be recognized and treated in a timely manner to prevent the long-term complications.

## Background

Graft-versus-host disease (GVHD) is a common complication of allogenic hematopoietic stem cell transplantation (HSCT) [1], the treatment widely used to treat a range of hematological malignancies such as leukemia and lymphoma. GVHD is an allogenic syndrome and a great mimicker of autoimmune diseases such as scleroderma, Sjogren’s syndrome, systemic lupus erythematosus, and rheumatoid arthritis [2]. It is an immunological phenomenon where donor cells initiate immune response, after engraftment, in the host (patient), as donor cells recognizes host tissue as foreign, thereby resulting in its development [3].

It is a multiorgan disease that more commonly affects skin, gastrointestinal tract, and liver [4], and less commonly affects lungs [5], kidneys [6], and haematopoietic system [7]. However, it is quite rare to involve the vascular system, particularly the larger vessels. Here, we describe a case of large-vessel vasculitis that occurred in a patient with chronic GVHD. After excluding the other causes, the vasculitis was ascribed to GVHD.

## Case presentation

A 59-year-old Caucasian man was diagnosed in 2016 with follicular lymphoma with diffuse large B-cell transformation when he presented with inguinal and cervical lymphadenopathy. He received six cycles of R-CHOP (rituximab plus cyclophosphamide, doxorubicin, vincristine, and prednisone) in September 2016 and remained in clinical remission for 18 months. He had a subclinical relapse of low-grade lymphoma (March 2018) with recurrent cervical lymphadenopathy and therefore received fludarabine and melphalan conditioned allogenic stem cell transplantation (Allo-HCT) from his human leukocyte antigen (HLA)-matched sibling (July 2019). Five months later, Allo-HCT was complicated by the development of classical multiorgan acute GVHD involving liver, skin, and gut (December 2019). He was then treated successfully with intravenous methylprednisolone 1 mg/kg daily for 3 days and switched to oral prednisolone and cyclosporin after symptoms settled. During ongoing follow-up, his cyclosporin was discontinued and prednisolone was tapered down to 12.5 mg daily, which is when he had an episode of isolated right-foot drop without systemic manifestations of GVHD or systemic vasculitis (April 2020). He was consulted by the neurology team and investigated with MRI lumbar spine and brain, which did not reveal any cerebrospinal pathology. However, this was not further investigated by nerve conduction study or other imaging modalities such as PET scan for the possibilities of systemic vasculitis as a causative factor for foot drop. Over a period of 8–10 weeks, his foot drop resolved spontaneously whilst the prednisolone was discontinued completely.

Three months later (August 2020), he developed recurrence of GVHD involving skin and gastrointestinal tract that manifested as diffuse erythematous rash and diarrhea without abdominal pain, and abdominal examination was unremarkable. Neurological examination was also unremarkable, including normal power in his lower limbs indicating complete resolution of his foot drop. His blood tests showed CRP 52 mg/L, ESR 73 mm/h, and autoimmune screen including ANA and ANCA were essentially unremarkable. Additionally, whole-body FDG CT/PET scan was performed to screen for recurrence of lymphoma, which showed mild increase in tracer activity in the ascending and descending aorta, and similar tracer activity in the subclavian, iliac, femoral, and popliteal arteries bilaterally suggestive of large-vessel vasculitis. There was no uptake or tracer activity on PET scan performed in 2017. For GVHD, he was treated with intravenous methylprednisolone 2 mg/kg followed by high-dose oral prednisolone and budesonide with steroid tapering plan at the time of discharge.

## Discussion

Vasculitis is an inflammation of blood vessels characterized by the presence of inflammatory leukocytes in the vessel wall. It occurs in the context of rheumatic and autoimmune disease, drugs, infections, and malignancies. Vasculitis is classified according to the size of blood vessels involved and the associated clinical features. Large-vessel vasculitis (LVV) often refers to vasculitis involving large- and medium-sized blood vessels, such as aorta and its branches [8]. Graft-versus-host disease is an immunological phenomenon where donor cells initiate immune response, after engraftment, in the host (patient), as donor cells recognizes host tissue as foreign, thereby resulting in the development of GVHD.

Both large-vessel vasculitis and GVHD are immunological phenomena. In LVV, the inflammation is mediated by dendritic cells, autoreactive T cells, macrophages, and histiocytes [9] [10], whereas in GVHD, the donor T-cell activation against host tissues leads to the development of disease [11]. Although the origin of the activated immune cells is different between LVV and GVHD, their effect on initiation of inflammation is similar. However, it is unclear why the larger vessels are targeted in particular, and association of LVV is rare in GVHD.

GVHD causing vasculitis has been described previously in brain, where it was shown to cause cerebral vasculitis. The vasculitis was detected on MRI brain when patients developed neurological symptoms, and vascular damage such as bleeding, aneurysms [12], brain atrophy, and ischemia was also detected [13]. However, the majority of these cases reportedly involve medium-to-smaller vessels. During our literature search, we encountered only one case of GVHD with LVV involving aorta and its branches, and in that case the vasculitis was attributed to GVHD.

Several studies have shown the association of vasculitis and various malignancies [14] 15. An incidental finding of LVV on PET scan has been demonstrated in half the patients who were being monitored for malignancies [15]. In another study, the successful treatment of malignancies was associated with the resolution of vasculitis, suggesting vasculitis is truly a paraneoplastic phenomenon [16]. In our patient, an episode of foot drop (mononeuritis multiplex) occurred while the patient was being weaned off prednisolone, and LVV was observed on PET scan with recurrent GVHD, together suggesting that GVHD was more likely to be related to a vasculitic process than a paraneoplastic. This is supported by resolution of LVV with treatment as observed on a repeat PET scan conducted at a 3-monthly interval (to confirm).

## Conclusion

To the best of our knowledge, our case is the second case of LVV in GVHD that has ever been reported in literature. The life-threatening vascular complications associated with LVV include arterial aneurysms and dissections, and ischemic or hemorrhagic stroke. Thus, this rare immunological association needs to be recognized and treated in a timely manner to prevent the long-term complications of LVV.

## Data Availability

The data analyzed during the current study are not publicly available due to patient confidentiality but are available from the corresponding author on reasonable request.

## Abbreviations

GVHD: Graft-versus-host disease
HSCT: Hematopoietic stem cell transplantation
HLA: Human leukocyte antigen
MRI: Magnetic resonance imaging
PET: Positron emission tomography
CRP: C-reactive protein
ESR: Erythrocyte sedimentation rate
ANA: Antinuclear antibody
ANCA: Antineutrophil cytoplasmic antibodies
FDG: Fluorodeoxyglucose
CT: Computed tomography
LVV: Large-vessel vasculitis
